# Comparative Effects of Sulforaphane and Allyl Isothiocyanate on 3T3-L1 Adipogenesis

**DOI:** 10.1155/2022/8705163

**Published:** 2022-01-19

**Authors:** Satoru Sakuma, Kana Yasuda, Risa Kitahara, Kaho Tsujimoto, Kishiko Yamashita, Naohiro Hoshino, Yohko Fujimoto, Keiichiro Okuhira

**Affiliations:** Department of Environment and Health Sciences, Faculty of Pharmacy, Osaka Medical and Pharmaceutical University, 4-20-1 Nasahara, Takatsuki, Osaka 569-1094, Japan

## Abstract

Sulforaphane and allyl isothiocyanate, naturally occurring isothiocyanates, have been reported to inhibit adipocyte differentiation, but little is known about how they compare in terms of their potency and mechanism of action. In the present study, we compared the effects of sulforaphane and allyl isothiocyanate on the differentiation of 3T3-L1 preadipocytes. A mixture of insulin, dexamethasone, and 3-isobutyl-1-methylxanthine was used to establish a differentiation medium. We found that, at a concentration as low as one-tenth that of allyl isothiocyanate, sulforaphane reduced triacylglycerol levels, lipid-filled adipocyte quantity, and mRNA and protein levels of CCAAT-enhancer-binding protein *α* (C/EBP*α*) and peroxisome proliferator-activated receptor *γ* (PPAR*γ*). These results suggested that sulforaphane may be a more potent adipocyte differentiation inhibitor than allyl isothiocyanate. Our results may provide insight into possible strategies for the prevention of obesity and related conditions.

## 1. Introduction

Obesity is a serious health problem worldwide, increasing the morbidity and mortality rates associated with several acute and chronic diseases, such as dyslipidemia, hypertension, type-2 diabetes, and cardiovascular disease [1, 2]. Obesity is characterized by increases in adipocyte quantity (hyperplasia) and size (hypertrophy) [3], which are regulated by genetic, metabolic, and nutritional factors [4]. Therefore, deciphering the mechanism by which certain nutrients affect adipocyte differentiation is important for preventing obesity and related conditions.

Isothiocyanates (ITCs) include both naturally occurring substances, such as sulforaphane and allyl isothiocyanate, and synthetically produced substances, such as fluorescein isothiocyanate and p-bromophenyl isothiocyanate. ITCs exhibit a wide range of known biological effects [5, 6]. These effects are derived from the reactivity of the ITC group (−NCS) and from the physicochemical properties of the non-ITC portion of the molecule, such as lipophilicity, shape, size, and rigidity. The reactivity of the ITC group determines the ability of the ITC to react with the functional groups of small biochemical molecules or biopolymers [7], and the physicochemical properties of the rest of the molecule may be responsible for its bioavailability in different cell compartments and tissues [8].

**Figure 1:**
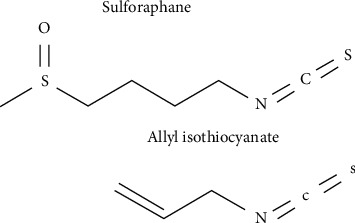
Structures of sulforaphane and allyl isothiocyanate.

Sulforaphane (Figure 1) is found in cruciferous vegetables such as broccoli sprouts, Brussels sprouts, and cabbage. Allyl isothiocyanate (Figure 1) is known to be responsible for the characteristic pungent aroma of some cruciferous vegetables, including radishes and horse radishes. Both of these are naturally occurring ITCs repress adipogenesis [9–11]. However, with the exception of their ITC moieties, the pharmacological differences caused by their distinct structures—the sulfinyl groups in sulforaphane and the allyl groups in allyl isothiocyanate—have not been investigated.

The present study investigated the comparative effects of sulforaphane and allyl isothiocyanate on triacylglycerol (TG) levels, lipid-filled adipocyte quantity, and mRNA and protein expression levels of CCAAT-enhancer-binding protein *α* (C/EBP*α*) and peroxisome proliferator-activated receptor *γ* (PPAR*γ*) during the differentiation of 3T3-L1 preadipocytes into mature adipocytes.

## 2. Materials and Methods

### 2.1. Materials

Mouse 3T3-L1 preadipocytes were obtained from the European Collection of Cell Cultures (Wiltshire, UK). The Transcriptor First Strand cDNA Synthesis Kit and LightCycler FirstStart DNA Master^plus^ SYBR Green reagent were obtained from Roche Diagnostics (Indianapolis, IN, USA). TRIzol reagent and the primers for *β*-actin, PPAR*γ*, and C/EBP*α* were purchased from Invitrogen (Carlsbad, CA, USA). Rabbit polyclonal anti-human *β*-actin, PPAR*γ*, and C/EBP*α* antibodies and goat HRP-linked anti-rabbit IgG antibody were obtained from Cell Signaling Technology, Beverly, MA, U.S.A. Sulforaphane, allyl isothiocyanate, and a protease inhibitor cocktail were obtained from Sigma Chemical Co. (St. Louis, MO, USA). The Triglyceride E-test Wako kit was obtained from Wako Pure Chemical Industries, Ltd. (Osaka, Japan). The Lipid Droplets Fluorescence Assay Kit was obtained from Cayman Chemical Co. (Ann Arbor, MI, USA). All other reagents used were of analytical grade.

### 2.2. Cell Culture

Mouse 3T3-L1 preadipocytes were cultured in Dulbecco's Modified Eagle's Medium supplemented with 10% fetal bovine serum and 1% penicillin-streptomycin (growth medium) at 37°C in a humidified atmosphere of 5% CO_2_/95% air. Cell differentiation was induced according to the protocol obtained from the European Collection of Cell Cultures. The procedure was initiated 2 d after the cells reached confluence. The cells were cultured for 3 d in differentiation medium (DM) containing 0.25 *µ*M dexamethasone, 0.5 mM 3-isobutyl-1-methylxanthine (IBMX), and 1 *µ*g/mL insulin. Next, the cells were cultured for 2 d in maturation medium containing 1 *µ*g/mL insulin. Finally, they were cultured in growth medium again for 2 d. ITC treatment, Oil Red O staining, and the determination of TG levels, lipid-filled adipocyte numbers, and mRNA and protein expression levels of PPAR*γ* and C/EBP*α* were performed according to our previously reported methods [12–14].

### 2.3. Treatment with Sulforaphane or Allyl Isothiocyanate

Sulforaphane and allyl isothiocyanate were prepared in dimethyl sulfoxide (Me_2_SO) and added to the growth, differentiation, and maturation media on day 3 (when dexamethasone, IBMX, and insulin were added). The Me_2_SO concentration was maintained at 0.25% of the total volume of the medium. Preliminary experiments demonstrated no significant effects of 0.25% *v*/*v* Me_2_SO on cell differentiation.

### 2.4. Oil Red O Staining

The cells were fixed in 4% formaldehyde phosphate buffer (pH 7.4) for 1 h, rinsed with water, and stained with 0.3% Oil Red O dye for 1 h. After washing again with water, the cells were observed under a microscope at 10 × 20 magnification.

### 2.5. Quantification of Mature Adipocytes

Mature adipocytes were quantified using the Lipid Droplets Fluorescence Assay Kit and flow cytometry. The cells were trypsinized carefully and centrifuged at 200 ×g for 5 min at 4°C. The cell pellet was resuspended, fixed, and stained with the lipophilic fluorescent dye Nile Red according to the manufacturer's instructions. The samples were then analyzed using a BD FACSAria III flow cytometer (Becton Dickinson, Basel, Switzerland). After excitation with a 488 nm argon ion laser source, Nile Red fluorescence was measured on the FL2 (FITC-A) emission channel through a 585 ± 21 nm bandpass filter. Using a forward scatter/side scatter representation, the P1 region was defined to exclude cellular debris. A P2 selection window was defined as the area with high FL2 (FITC-A) values, representing mature adipocytes in the FL2 (FITC-A)/cell count blot of the P1 population, as described by Sottile and Seuwen [15]. Data analysis was performed using BD FACSDiva software (ver. 8.0, Becton Dickinson, Franklin Lakes, NJ, USA). For each sample, 20,000 events were recorded, and results were expressed as the percentage of cells in the P2 region.

### 2.6. Determination of TG Levels

The cells were harvested by scraping them from the culture dishes into a lysis buffer of 1% Triton-X100, 150 mM NaCl, 4 mM ethylenediaminetetraacetic acid, and 20 mM Tris-HCl (pH 7.4) containing a protease inhibitor cocktail. Cells were lysed completely using a horn-type sonicator. TG level, a lipid accumulation index, was determined using the Triglyceride E-test Wako kit after protein level normalization and was expressed as TG content (*µ*g/mg protein).

### 2.7. Determination of the mRNA Expression Levels of *β*-actin, PPAR*γ*, and C/Ebp*α*

At day 5, untreated cells were used as a control. Cells treated with sulforaphane or allyl isothiocyanate were washed with ice-cold phosphate-buffered saline. Total cellular RNA was extracted using TRIzol reagent, and 1 *μ*g of total RNA was reverse transcribed into cDNA using the Transcriptor First Strand cDNA Synthesis Kit. The concentration and quality of the purified total RNA were determined spectrophotometrically at 260 nm. The ratio of optical density at 260 nm to that at 280 nm was also calculated. Next, mRNA expression was determined by real-time reverse transcription polymerase chain reaction using the SsoAdvanced^TM^ SYBR® Green Supermix (Bio-Rad, Hercules, CA, USA) reagent and a CFX Connect™ instrument (Bio-Rad). The results were expressed as the target mRNA level relative to that of *β*-actin mRNA, and the values obtained in the presence or absence of the drugs were expressed relative to the values associated with exposure to the DM alone.

The primers used were as follows: *β*-actin, 5′-ACACCCCAGCCATGTACG-3′ and 5′-TGGTGGTGAAGCTGTAGCC-3′, PPAR*γ*, 5′-GTGAAGCCCATCGAGGACA-3′ and 5′-TGGAGCACCTTGGCGAACA-3′, and C/EBP*α*, 5′-ATGGTTTCGGGTCGCTGGAT-3′ and 5′-CCACGGCCTGACTCCCTCAT-3′.

### 2.8. Determination of the Protein Expression Levels of *β*-actin, PPAR*γ*, and C/Ebp*α*

Untreated or sulforaphane or allyl isothiocyanate-treated 3T3-L1 cells up to day 5 were washed with ice-cold PBS and lysed. An equal volume of solubilization buffer (20% glycerol, 4% SDS, 2% 2-mercaptoethanol, 125 mM Tris/HCl, pH 6.8) was added and the mixture was boiled for 10 min. Cell lysates were analyzed using a 7.5% polyacrylamide gel. Proteins were transferred to nitrocellulose membranes by electroblotting, and the membranes were incubated overnight in TBS-T (0.14 M NaCl, 20 mM Tris, 0.1% Tween 20, pH 7.4) containing their respective primary antibodies (*β*-actin, PPAR*γ*, and C/EBP*α* antibodies) and 3% skimmed milk powder. The membranes were incubated overnight. After incubation, the membranes were incubated with secondary goat HRP-conjugated anti-rabbit IgG antibody for 1 h, followed by Amersham ECL Western blotting detection reagent (Cytiva, Marlborough, MA, U.S.A.).

### 2.9. Statistical Analysis

The results are expressed as the mean ± standard error. Significant differences in data between the two groups were assessed using Student's *t*-test, and differences between multiple groups were assessed using one-way analysis of variance followed by Scheffé's multiple range test. Differences were considered statistically significant at *P* < 0.05.

## 3. Results

### 3.1. Effects of Sulforaphane and Allyl Isothiocyanate on 3T3-L1 Cell Differentiation

**Figure 2:**
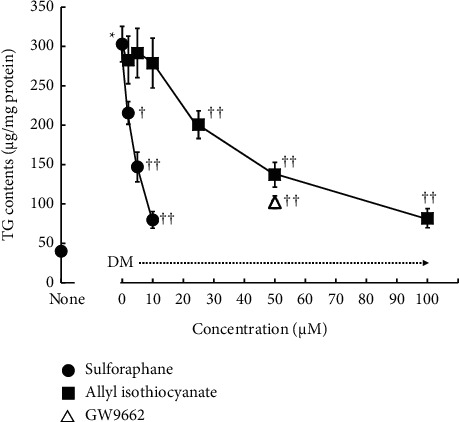
Alteration in triacylglycerol (TG) levels in 3T3-L1 adipocytes treated with sulforaphane or allyl isothiocyanate. Data are presented as the mean ± standard error (*n* = 4−16). ^*∗*^denotes *P* < 0.01, versus untreated cells (none); ^†^denotes *P* < 0.05; ^††^denotes *P* < 0.01, versus cells cultured in differentiation medium.

**Figure 3:**
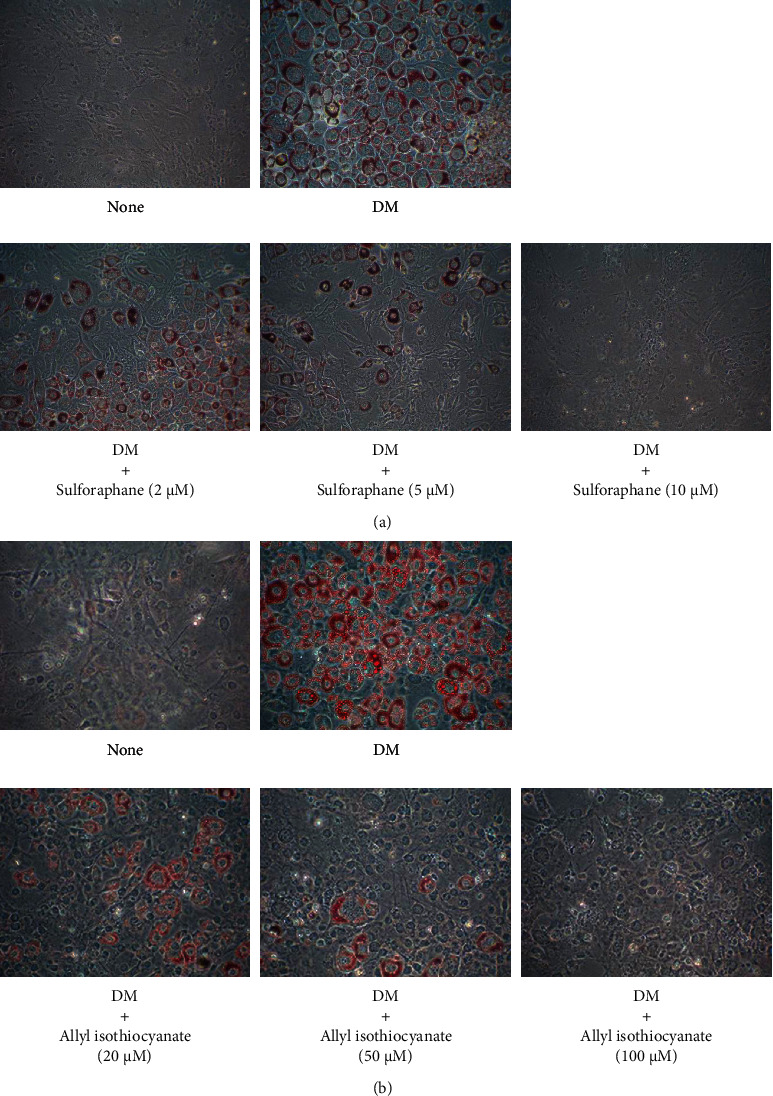
Results of Oil Red O staining of 3T3-L1 adipocytes treated with sulforaphane (a) or allyl isothiocyanate (b), representative images from six independent experiments.

**Figure 4:**
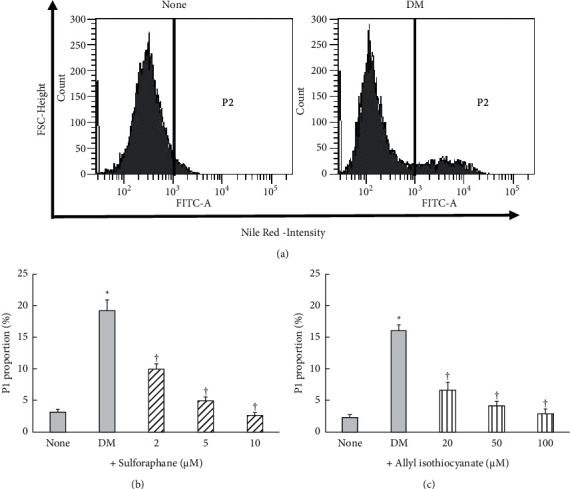
Results of Nile Red staining and flow cytometry analysis of 3T3-L1 adipocytes treated with sulforaphane (b) or allyl isothiocyanate (c). (a) Representative images showing results of the flow cytometry analysis using Nile Red staining. Data are presented as the mean ± standard error (*n* = 5). ^*∗*^denotes *P* < 0.01; ^†^denotes *P* < 0.01, versus untreated cells (none) or cells cultured in differentiation medium (DM), respectively. FSC denotes forward scatter.

Figure 2 displays the effects of sulforaphane and allyl isothiocyanate on TG accumulation in 3T3-L1 adipocytes. The TG level of DM-cultured cells was approximately 8 times higher than that of the control cells. Sulforaphane decreased TG content in a concentration-dependent manner, with concentrations of 2–10 *µ*M inducing 28%–74% reductions. Allyl isothiocyanate induced 32%–73% decreases in TG content at concentrations of 25–100 *µ*M, at least 10 times the concentrations of sulforaphane required for the same effect. A 50 *µ*M concentration of GW9662 [16], a PPAR*γ* antagonist, reduces TG accumulation by 66%. Thus, allyl isothiocyanate suppressed TG content at nearly the same rate as GW9662, while sulforaphane's inhibitory effect was much stronger. As shown in Figures 3 and 4, the DM-cultured cells exhibited larger quantities of Oil Red O-stained cells (Figure 3) and lipid-filled adipocytes (Figure 4) than the control cells. Compared to cells that were cultured in DM alone, DM-cultured cells treated with 2, 5, or 10 *µ*M sulforaphane exhibited a concentration-dependent reduction in fat droplet quantity (Figure 3(a)) and a significant decrease—48%, 74%, and 86%, respectively—in the quantity of lipid-filled adipocytes (Figure 4(b)). In contrast, treatment of DM-cultured cells with 20, 50, or 100 *µ*M allyl isothiocyanate resulted in concentration-dependent reduction of the quantities of Oil Red O-stained cells (Figure 3(b)) and in, respectively, 59%, 74%, and 83% reductions in lipid-filled adipocytes (Figure 4(c)). These rates were slightly lower than those observed in the cells treated with sulforaphane, at approximately 10 times the concentration.

### 3.2. Effect of Sulforaphane and Allyl Isothiocyanate on PPAR*γ* and C/Ebp*α* mRNA Expression Levels

**Figure 5:**
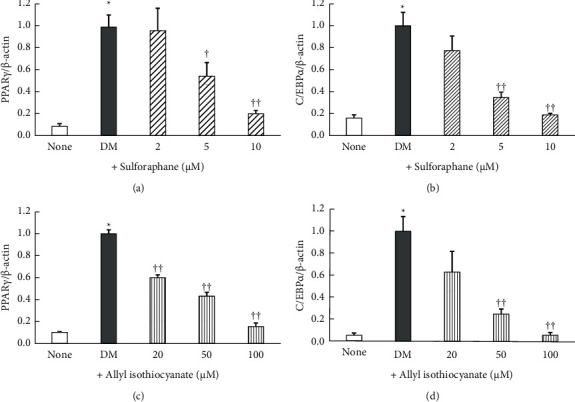
Peroxisome proliferator-activated receptor *γ* (PPAR*γ*) and CCAAT-enhancer binding protein *α* (C/EBP*α*) mRNA expression levels in 3T3-L1 adipocytes treated with (a, b) sulforaphane or (c, d) allyl isothiocyanate. Data are presented as the mean ± standard error (*n* = 3-4). ^*∗*^denotes *P* < 0.01, versus untreated cells (none); ^†^denotes *P* < 0.05; ^††^denotes *P* < 0.01, versus cells cultured in differentiation medium (DM).

As shown in Figure 5, the DM-cultured cells exhibited markedly higher PPAR*γ* and C/EBP*α* mRNA expression levels than the control cells, and the PPAR*γ* and C/EBP*α* mRNA expression levels were significantly (*P* < 0.05 or 0.01) suppressed by the addition of sulforaphane (Figures 5(a) and 5(b)) or allyl isothiocyanate (Figures 5(c) and 5(d)). Exposure to sulforaphane at concentrations of 5 and 10 *µ*M reduced PPAR*γ* mRNA expression levels by 45% and 80%, respectively, and C/EBP*α* mRNA expression levels by 65% and 81%, respectively. Exposure to allyl isothiocyanate at concentrations of 50 and 100 *µ*M reduced PPAR*γ* mRNA expression levels by 57% and 85%, respectively, and C/EBP*α* mRNA expression levels by 75% and 94%, respectively. Thus, sulforaphane had a similar inhibitory effect on C/EBP*α* and PPAR*γ* mRNA expression levels as allyl isothiocyanate, at a 10-fold lower concentration.

### 3.3. Effect of Sulforaphane and Allyl Isothiocyanate on PPAR*γ* Protein Levels

**Figure 6:**
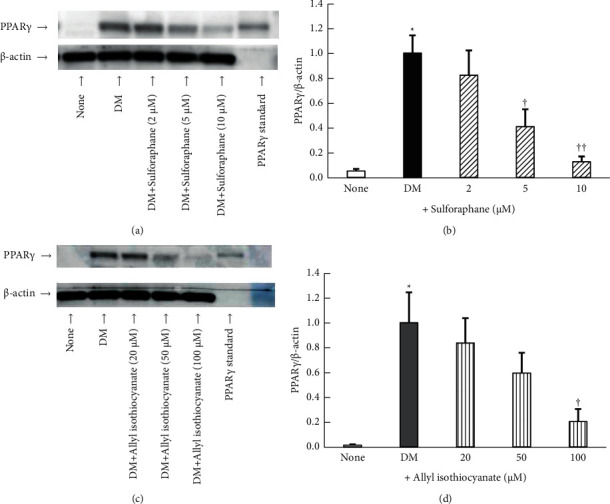
Peroxisome proliferator-activated receptor *γ* (PPAR*γ*) protein levels in 3T3-L1 adipocytes treated with (a, b) sulforaphane or (c, d) allyl isothiocyanate. Data are presented as the mean ± standard error (*n* = 4). ^*∗*^denotes *P* < 0.01, versus untreated cells (none); ^†^denotes *P* < 0.05; ^††^denotes *P* < 0.01, versus cells cultured in differentiation medium (DM).

As shown in Figure 6, the DM-cultured cells exhibited markedly higher PPAR*γ* protein levels than the control cells. PPAR*γ* protein levels were significantly (*P* < 0.05 or 0.01) suppressed by the addition of sulforaphane (Figures 6(a) and 6(b)). Exposure to 5 and 10 *µ*M concentrations of sulforaphane reduced PPAR*γ* protein levels by 59% and 87%, respectively (Figure 6(b)), while exposure to 100 *µ*M allyl isothiocyanate produced an 80% reduction (Figure 6(d)).

## 4. Discussion

The results of the present study clearly showed that while both sulforaphane and allyl isothiocyanate inhibited adipocyte differentiation, sulforaphane was a much stronger inhibitor. This suggests that the anti-obesity effect of sulforaphane is greater than that of allyl isothiocyanate. Furthermore, we demonstrated that the effects of both sulforaphane and ally isothiocyanate were associated with the suppression of mRNA expression levels, leading to the downregulation of PPAR*γ* protein levels.

The mechanism by which sulforaphane inhibits adipocyte differentiation more strongly than allyl isothiocyanate is unclear. Adipogenesis is correlated with various signaling pathways, such as insulin signaling and the PPAR regulation pathway, which are promising drug targets for obesity and metabolic disease treatment [17, 18]. The insulin receptor (IR) catalytic ability is dependent on IR expression levels and IR substrate 1 (IRS-1) tyrosine phosphorylation. IR signaling leads to serine/threonine protein kinase B (Akt/PKB) activation, which subsequently activates the C/EBP*α*-PPAR*γ* pathway. PPAR*γ* functions as a master regulator of adipocyte differentiation, whereas C/EBP*α* works with PPAR*γ* to induce adipocyte differentiation. Nuclear factor erythroid factor 2 (Nrf2) activators have been shown to improve insulin signaling and decrease adipose differentiation through multiple mechanisms [19]. However, Ernst et al. [9] reported that treatment with sulforaphane and other ITCs, such as allyl isothiocyanate, results in comparative levels of phase 2 enzymes including *γ*-glutamyl cysteine synthetase and antioxidant enzymes via an Nrf2-dependent signal transduction pathway in cultured cells and *in vivo* experiments. Conversely, Valli et al. [10] and Xu et al. [11] demonstrated that sulforaphane-treated obese mice exhibited significantly increased IRS-1 protein levels and marked Akt/PKB activation. Comparison of IRS-1 upregulation by sulforaphane and other ITCs, including allyl isothiocyanate, will require additional investigation. Most importantly, the difference in the mechanism of action of the two compounds in inhibiting adipogenesis not only is the difference in the expression of C/EBP*α* and PPAR*γ* but also requires further research.

The logP, or the water and *n*-octanol partition coefficient, is 0.23 ± 0.39 for sulforaphane and 1.77 ± 0.30 for allyl isothiocyanate [20], indicating that sulforaphane is more hydrophilic than allyl isothiocyanate. This is likely due to differences in the physicochemical properties of the side chains, excluding the ITC group [7, 8], and indicates that the nonspecific passage of sulforaphane into the cell is less favorable than that of allyl isothiocyanate; instead, sulforaphane may bind specifically to certain proteins expressed on the cell surface. Research on membrane proteins that bind specifically to sulforaphane may yield additional clarity.

## 5. Conclusion

Sulforaphane, a potent antioxidant derived from glucosinolates in cruciferous vegetables, has been identified as a promising chemopreventive agent to combat cancer [21–23]. Of course, the present in vitro results using 3T3-L1 cells may or may not be replicated in vivo. However, the results of this study suggest that, in addition to its known bioactivity, sulforaphane may contribute to the prevention of lifestyle-related diseases by conferring a protective effect against obesity. Our future plans include investigating sulforaphane's mechanism of action by examining its structure-activity relationship, as well as studying the medicinal effects of its derivatives, which we hope will contribute to the discovery of preventive treatments for obesity and lifestyle-related diseases to further the maintenance and promotion of health.

## Data Availability

The data used to support the findings of this study are available from the corresponding author upon request.
